# Personality, Health Care Use, and Costs: A Study Protocol for a Systematic Review

**DOI:** 10.3390/healthcare8030263

**Published:** 2020-08-12

**Authors:** André Hajek, Benedikt Kretzler, Hans-Helmut König

**Affiliations:** Department of Health Economics and Health Services Research, University Medical Center Hamburg-Eppendorf, 20246 Hamburg, Germany; b.kretzler.ext@uke.de (B.K.); h.koenig@uke.de (H.-H.K.)

**Keywords:** personality, health care use, health services research, primary care, GP visits, neuroticism, systematic review

## Abstract

Recent empirical studies have shown that personality factors are associated with health care use (HCU). However, to date, a systematic review is lacking summarizing evidence regarding the link between personality factors and health care use or costs (i.e., monetarily valued health care use). Therefore, the objective of this systematic review is to provide an overview of evidence from observational studies investigating the link between personality characteristics and health care use or costs. Electronic databases (PubMed, PsycINFO, NHS EED) will be searched using predefined search terms. In an additional step, the reference lists of included studies will be searched (manually). No restrictions will be applied regarding the time of publication. Observational studies (both cross-sectional and longitudinal) assessing the link between personality characteristics and health care use/costs across all age categories will be included. Only studies using validated tools to quantify personality characteristics will be included. Among others, studies only focusing on mental HCU or studies only analyzing samples with a specific disorder (e.g., individuals with personality disorders) will be excluded. Mainly, data on methods (study design, measures, and statistical analysis), sample characteristics, and results regarding the link between personality and HCU/costs will be extracted. A quality assessment will be conducted. Two reviewers will perform the study selection, data extraction, and assessment of the study quality. If disagreements occur, they will be resolved through discussion to reach a consensus or by inclusion of a third party. Results will be presented narratively (text and tables). Depending on the number and heterogeneity of the studies included, a meta-analysis will be conducted. Results will be disseminated through publication in a peer-reviewed, scientific journal.

## 1. Introduction

Understanding the determinants of health care use (HCU) is important to manage health care resources (e.g., to avoid misuse or overuse). Based on the Andersen behavioral model [1,2], numerous studies have investigated the determinants of HCU (e.g., [3,4,5,6,7,8,9]). This model distinguishes between (i) predisposing characteristics like sex or age, (ii) enabling resources like income, and (iii) need factors, such as self-rated health or chronic illnesses. According to a previous systematic review, HCU is mainly driven by need factors [10]. However, some recent empirical studies have demonstrated that personality characteristics are also important for HCU [11,12]. For example, Friedman et al. [11] showed that neuroticism was associated with HCU in the United States cross-sectionally. Furthermore, they showed a link between higher openness to experience, higher agreeableness, lower conscientiousness, and HCU. Additionally, in another study [12], it has been shown that neuroticism was associated with increased physician visits in Germany longitudinally. Furthermore, extraversion was associated with an increased likelihood of hospitalization [12]. A recently published article also stressed the need for studies examining the link between personality factors and HCU [13].

Commonly, personality is divided into five traits [14]: (1) agreeableness (tendency to get along well with others), (2) conscientiousness (extent to which an individual is persevering, reliable, and careful), (3) extraversion (tendency to have a positive outlook in life and to experience positive feelings), (4) neuroticism (tendency to experience negative emotions, such as anger or anxiety), and (5) openness to experience (tendency to be imaginative or open-minded).

As stated, some recent empirical studies have demonstrated that personality factors such as extraversion or neuroticism are associated with HCU (for example, [11,12]). However, as yet, there is a lack of studies that systematically synthesize studies investigating personality factors and HCU or costs. Consequently, the purpose of this study is to give an overview of evidence from observational studies investigating the link between personality characteristics and HCU or costs.

Furthermore, it is worth noting that a study protocol for a systematic review is an important component of the whole systematic review process. For example, it ensures that the systematic review is planned carefully. Moreover, it ensures that the process is explicitly documented before starting the review [15]. This promotes research integrity, accountability, and transparency of the finished review [15]. Additionally, it can help to anticipate problems, and it can enable readers to determine differences between planned and used methods in the final reviews, which, in turn, can help to identify potential biases [15].

## 2. Materials and Methods

This protocol for our systematic review was done taking into consideration the Preferred Reporting Items for Systematic Reviews and Meta-Analysis Protocols guidelines [15]. Our study is registered with the International Prospective Register of Systematic Reviews (PROSPERO, registration number: CRD42020170800).

### 2.1. Eligibility Criteria

The inclusion/exclusion criteria are presented in the next sections. Based on these criteria, studies will be considered for inclusion. A pretest will be conducted (prior to final eligibility criteria) using a sample of 100 titles/abstracts. If required, eligibility criteria will be refined.

#### 2.1.1. Inclusion Criteria

Inclusion criteria will be as follows:

- Observational studies (both cross-sectional and longitudinal) assessing the link between personality characteristics and HCU/costs across all age categories.
- Studies using validated tools to quantify personality characteristics.
- Publications in English or German published in peer-reviewed journals.

#### 2.1.2. Exclusion Criteria

Exclusion criteria are as follows:

- studies not reporting the association between personality characteristics and HCU or costs;
- studies only focusing on mental HCU;
- studies only analyzing samples with a specific disorder (e.g., individuals with personality disorders);
- study design other than observational;
- assessment of personality or HCU not appropriate (e.g., not using validated tools to assess personality or quantifying HCU without providing details regarding the observation period);
- studies not published in a peer-reviewed journal or in a language other than German or English.

Several electronic databases (PubMed, PsycINFO, NHS EED) will be used for identifying studies in March 2020. Prior to the submission of the systematic review, it is planned to update the search.

Predefined search terms (e.g., personality) will be used. In Table 1, the PubMed search strategy is displayed. Restrictions do not apply with regard to location or time of the publication. If required, search terms will be adapted to each specific database. Furthermore, the two reviewers will examine the reference lists of the included articles to identify additional relevant articles (i.e., articles that met our eligibility criteria).

### 2.2. Data Management

All articles will be imported in EndNote X7 (Clarivate Analytics (formerly Thomson Reuters), Philadelphia, PA, USA). If a meta-analysis is possible, Stata 16.0 (StataCorp, College Station, TX, USA) will be used for conducting it.

### 2.3. Study Selection Process

After the electronic and manual search is done, all studies will be evaluated for inclusion/exclusion in a two-step process (independently performed by two reviewers (A.H., B.K.) using defined selection criteria):

1. Title/abstract screening.
2. Full-text screening.

Disagreements between the two reviewers will be resolved through discussion to reach consensus or by inclusion of a third party (H.-H.K.). This procedure regarding disagreement will also be used for the data collection process and assessment of study quality/risk of bias.

### 2.4. Data Collection Process and Data Items

Data extraction will be conducted by two reviewers (A.H. and B.K.). While one reviewer will extract important data from the studies, the second reviewer will conduct a cross-check. When important data cannot be extracted or clarification is required, the authors of the respective studies will be contacted.

Data on study design, definition, and assessment of key variables, sample characteristics, statistical analysis, and results with regard to the link between personality characteristics and HCU or costs will be extracted.

### 2.5. Assessment of Study Quality/Risk of Bias

The quality of the studies will be assessed using an appropriate quality assessment tool for HCU and COI studies (primarily according to the “Checklist for the Development and Assessment of Cost-of-Illness Studies” developed by Müller et al. [16]). The quality assessment will be performed independently by two reviewers (A.H. and B.K.). Results from the quality assessment will be included in the synthesis of results.

### 2.6. Data Synthesis

A PRISMA flowchart will be used to display the study selection process. The results from the data extraction and quality assessment will be synthesized narratively in text and tables. If possible, results will be categorized according to personality trait analyzed (openness to experience; conscientiousness; extraversion; agreeableness; neuroticism) or outcome (e.g., outpatient; inpatient). If appropriate, a meta-analysis will be performed to obtain a quantitative synthesis of the results.

### 2.7. Patient and Public Involvement Statement

The present review protocol did not involve individual patients or public agencies.

## 3. Discussion

To date, there is a lack of systematic reviews investigating the association between personality characteristics and HCU or costs. This systematic review will provide an overview of evidence from observational studies investigating the link between personality characteristics and HCU or costs across all age categories. Furthermore, the quality of included studies will be evaluated. For example, differences in statistical methods (e.g., different regression techniques) may be identified. If possible, a meta-analysis will be performed.

### Strenghs and Limitations

This will be the first systematic review synthesizing and critically assessing evidence from longitudinal and observational studies on the link between personality, HCU, and costs. Using observational data (both cross-sectional and longitudinal) and samples that are not illness-specific (like individuals with personality disorders) may produce widely generalizable results. Furthermore, two independent reviewers are involved in the processes of study selection, data extraction, and quality assessment. A potential limitation is that conducting a meta-analysis may not be appropriate due to the possible heterogeneity between studies.

## 4. Conclusions

Our systematic review could stress potential gaps in research (for example, a general lack of studies or a lack of longitudinal studies investigating the link between personality characteristics and HCU or costs) and quality of recent studies. Our systematic review may also assist in managing HCU.

## Figures and Tables

**Table 1:** Search query (PubMed).

#	Search Term
#1	Personality [Title/Abstract]
#2	Big five [Title/Abstract]
#3	#1 OR #2
#4	Health care
#5	Health service *
#6	#4 OR #5
#7	Use
#8	Utili *
#9	#7 OR #8
#10	#6 AND #9
#11	cost
#12	expense *
#13	expenditure *
#14	economic *
#15	#11 OR #12 OR #13 OR #14
#16	#10 OR #15
#17	#3 AND #16

Note: The asterisk (*) is a truncation symbol. The number sign (#) refers to the search order.
